# Framework and strategy for integrated monitoring and evaluation of child health programmes for responsive programming, accountability, and impact

**DOI:** 10.1136/bmj.k2785

**Published:** 2018-07-30

**Authors:** Theresa Diaz, Kumanan Rasanathan, Emmanuel Meribole, Isabella Maina, Humphreys Nsona, Kyaw Myint Aung, Bennett Nemser, Kathryn Patricia O’Neill

**Affiliations:** 1 Epidemiology and Monitoring and Evaluation Team, Maternal Newborn and Child Adolescent Health Department, World Health Organization, Geneva, Switzerland; 2 Knowledge Management and Implementation Research Unit, Health Section, Unicef, New York, USA; 3 Monitoring and Evaluation, Nigeria Ministry of Health, Abuja, Nigeria; 4 Health Sector Monitoring and Evaluation Unit-MOH;,Nairobi, Kenya; 5 Integrated Management of Childhood Illnesses, Ministry of Health, Malawi; 6 Unicef, Malawi Lilongwe, Malawi; 7 Global Platform on Measurement and Accountability, Department of Information, Evidence and Research, Health Systems and Innovation, World Health Organization, Geneva, Switzerland

## Abstract

Stronger governance and leadership is needed to implement and maintain integrated reporting systems, say **Theresa Diaz and colleagues**

Monitoring and evaluation of health systems, programmes, and interventions is critical to assess progress, identify problems, and facilitate change to improve service delivery and reach the desired outcomes.^1^ ^2^ ^3^ Funders increasingly demand monitoring and evaluation so that they can determine whether a programme achieves its intended outcomes.

As the recognition and importance of monitoring and evaluation has grown so has the push towards integration of health services. For the sustainable development goals adopted by countries at the United Nations in 2015, the global health policy pendulum is swinging towards the need for “integration” of health service delivery to achieve universal health coverage (which is one of the sustainable development health targets).^4^ However, less attention has been paid to the consequent need for monitoring and evaluation systems to be integrated, through design and implementation that allows the measurement and analysis of multiple outputs and outcomes in a single report.

An integrated strategy for management of childhood illness has been in place for 20 years. The Integrated Management of Childhood Illness (IMCI) was developed by the World Health Organization and Unicef in 1995. Its aim was to reduce child mortality and improve child health in countries with a mortality rate in the under 5s of >40/1000. It tackles multiple child illnesses through integrated delivery of preventive and curative services.^5^ More than 100 countries have adopted the IMCI strategy for their health programmes and systems. Its three components—improving health worker skills, strengthening health systems, and improving family and community practices—have been adopted to a varying extent.^6^ However, there have been challenges to creating and sustaining a monitoring and evaluation system to support the programme.

Here we describe the challenges created by the fragmentation of the current monitoring and evaluation systems and suggest solutions. We have used data mainly from the IMCI implementation survey,^6^ interviews with key informants and in-depth country assessments carried out as part of the 2016 strategic review of IMCI.^7^

## Why monitoring and evaluation are not integrated

Between 1997 and 1999 an attempt was made to foster integration and harmonise indicators. An interagency working group on IMCI monitoring and evaluation recommended a standard set of indicators for use of IMCI at first level health facilities and in the community.^8^ This set of 24 indicators included health system indicators as well as nutrition, prevention (immunisation and bed net use to prevent malaria), and caretaker satisfaction. Further attempts to bring about integration included a guide for monitoring and evaluation of child health programmes in 2005.^9^ However, these recommendations failed to deal with the reality. Health management information systems (HMIS) are data collection systems specifically designed to support planning, management, and decision making. Often immunisation, HIV, malaria, and nutrition use different information systems. Similarly, civil registration and vital statistics systems and child death audits, which track births, deaths, and potential causes of death, use separate reporting forms and electronic reporting systems. These non-integrated systems for collection of routine data are in place because each vertical programme, globally and nationally, is funded, governed, and managed separately. In addition, IMCI monitoring and evaluation uses data from other sources. For example, data on exclusive breast feeding, use of bed nets, and very often mortality data require household population based surveys. Information on quality of care, experience of care, and availability of commodities requires health facility surveys. Pulling these multiple data sources together and conducting integrated analysis is complex and thus infrequently carried out.

### Lack of resources for integration

Efforts to support routine monitoring and evaluation systems have typically focused on a single programme, usually owing to availability of resources. For example, in Malawi 42 types of data tools were in use. Each technical programme also collects its own indicators, and data are stored in disparate systems (H Nsona, personal communication, 2017). In Nigeria, the well funded polio eradication programme has been given political priority, as have routine immunisation data. Both of these were allocated a module on the health management information systems platform (other programmes were not (E Meribole, personal communication, 2017)). Multiple single disease reporting systems often result in an individual healthcare worker reporting multiple programmes using multiple forms. This can be cumbersome, duplicative, time consuming and lead to poor quality of data.^10^ ^11^ Key informants felt that monitoring and evaluation should avoid an approach in which a global programme dictates what data need to be collected. An academic researcher in a high income country suggested that such an approach was unhelpful and “. . . more often than not leads to game playing and data tampering.” It was suggested that the role of WHO should be to encourage country offices to track a short list of key indicators.

Global and national investment into health monitoring and evaluation systems has been insufficient, with a notable lack of training, supervision, and funding for officers. This absence of investment can result in a reduced ability to report findings owing to a lack of supervision and insufficient tools to capture data. This was the case in India, where reporting was hampered by poor availability of forms and registers. Indicators to monitor programme performance were not included in the health management information systems, resulting in an adverse effect on the flow of information at district and state level.

The limited capacity to analyse data from multiple sources and use the findings hampers the provision of information that might improve the efficiency and quality of health service delivery. All these factors can lead to healthcare workers not recording data collected and not trusting the data captured. As one academic researcher based in a low income country suggests, “workers don’t have time to input the data and nobody trusts the data, so nobody uses them and, in turn, healthcare workers care even less about the data.”

### Global initiatives support parallel monitoring and evaluation

Child health programmes entail not only treatment of major causes of mortality such as pneumonia, malaria, and diarrhoea but also preventive services such as immunisations, provision of nutritional services, and prevention and treatment of HIV and tuberculosis (TB). Surveillance, monitoring, and evaluation of malaria, HIV, TB, nutrition, and immunisation are usually done through separate donor funded programmes, with global disease specific guidance and reporting forms and single disease monitoring systems. This has further increased fragmentation as shown by uncoordinated parallel data collection systems, analysis of multiple data sources focusing on only one disease, and the lack of resources provided to other non-donor funded monitoring and evaluation programmes.

More recently, in alignment with the sustainable development goals, a set of indicators and a monitoring and evaluation framework have been developed specifically for maternal, newborn, and child health,^12^ but large gaps in data remain.^13^ In part, this may be because these indicator frameworks are intended for global reporting. Some guidance is available for maternal, newborn, and child health programmes at subnational, facility, and community levels.^9^ ^14^ However, this needs to be updated to reflect new technologies, to better integrate facility and community reporting systems, and, most importantly, to align with data that are useful and needed locally to improve programmes.^14^ ^15^

### Weak national leadership and governance

Strong country leadership and clear governance are necessary to ensure that standardised key indicators are available in health management information systems and collected through surveys when needed. Globally, according to the IMCI implementation survey, monitoring has been relatively infrequent and inconsistent.^6^ Only 33% of countries (30/91) reported having a comprehensive IMCI monitoring and evaluation plan. Although more than two thirds of countries (70%; 66/94) reported that their health management information systems included some monitoring indicators for IMCI, only about one third of countries (34%; 32/95) in the past 5 years had conducted a health facility survey collecting data relevant to IMCI.

Several countries in this review highlighted the lack of national leadership and governance. India reported that IMCI indicators are not available in health management information systems. Kenya has no legal framework to enforce the use of standardised data tools or reporting (I Maina, personal communication, 2017). In Nigeria, separate funding sources, weak leadership and enforcement, and lack of ownership have led to vertical alignment within child health monitoring and evaluation programming (E Meribole, personal communication, 2017). Similarly, in Malawi each technical programme collects its own indicators (H Nsona, personal communication, 2017).

## Achieving integration

Future major goals are to reduce the burden on frontline health workers and improve the quality and use of data. This requires global action to synergise investments and requirements for reporting systems, national action to improve leadership and governance by establishing and requiring one reporting system for child health, and action at all levels to better use technology and support and build the ability of staff to collect and use data.

Some progress in alignment of global initiatives has been made. In 2015, WHO collaborated with 19 international and multilateral partners and countries to develop and agree on a global reference list of 100 core health indicators.^15^ The list reflects indicators of relevance for country, regional, and global reporting across the full spectrum of global health priorities, including IMCI. Building on the work of the 100 core health indicators, the Health Data Collaborative was created to provide a platform for countries, partners, and donors for better alignment of funding and technical support for health monitoring and evaluation systems.^16^ The aim is to contribute to reduced reporting requirements and to align investments in one common country monitoring and evaluation system.

Responsibility for introducing a national integrated child health monitoring and evaluation system must reside in national institutions with overall stewardship of monitoring and evaluation. These institutions are generally ministries of health, which must institute and enforce integrated data collection using one set of shared indicators and one child health reporting form for health management information systems, and supporting integrated analysis.^17^ ^18^ ^19^ Box 1 provides some examples of improved national governance and leadership.

Technology needs to be better used globally, nationally, and subnationally to improve the collection, analysis, and use of data. We found innovative technological solutions being employed in some countries. These included web based reporting systems in Bangladesh, Kenya, and Nigeria and real time data collection in the Democratic Republic of Congo using healthcare workers and local community volunteers (box 1), Globally, we suggest that an increased use of mobile health technologies, adoption of national standards for health management information systems (eg, *District Health Information System* 2),^20^ ^21^ ^22^ ^23^ as well as interoperability and integration of electronic reporting systems must be in place.^24^

Box 1 **Innovative national solutions to improving monitoring and evaluation**

*Bangladesh:* One major innovation used here is eIMCI, a web based management system for the Integrated Management of Childhood Illness (IMCI). Some key informants suggested that IMCI paved the way for improvement of health management information systems. “Web based management information systems were initially started for IMCI and are now used for everything.”
*Democratic Republic of Congo:* The country has a monitoring system, known as “improved monitoring for action.” This is used to track progress in effective coverage of maternal, newborn and child health interventions. Monitoring is carried out by health workers helped by volunteers from the communities being served. Efforts are now focusing on developing real time data collection and centralised transmission.

Finally, some of the approaches used for monitoring a specific disease may be adapted for integrated monitoring and evaluation. Specifically, methods should be adopted to improve the ability of health workers to perform monitoring and evaluation, such as regional workshops supplemented with online courses and participatory training.^25^ ^26^

**Fig 1:**
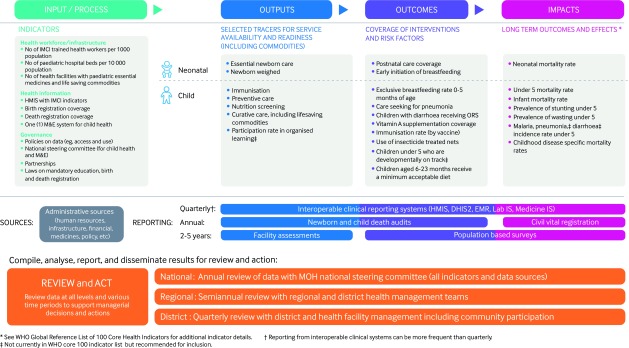
Monitoring and evaluation framework for integrated management of newborn and child illness. DHIS2=District Health Information System 2; EMR=electronic medical record; HMIS=health management information systems; IMCI=integrated management of childhood illness; Lab IS=Laboratory Information System; M&E=monitoring and evaluation; Medicine IS=Medicine Information System; MOH, Ministry of Health; ORS=oral rehydration solution

We propose a strategy for monitoring and evaluation of child health as shown in fig 1. 

We emphasise the need for a small set of key inputs, outputs, and outcomes and data sources for collecting common measurements and having the systems in place to collect such data. These include administrative data, interoperable health management information systems, facility and population based surveys, and fully functional civil registration and vital statistics supplemented by death audits—in particular, audits of newborn and child death. Using multiple data sources to analyse and act on findings is a complex process. We suggest increased use of electronic systems to link data sources and generate automatic reports and visualisations (eg, score cards) as well as regular workshops that bring stakeholders together to review data analysis and plan for action.

## Conclusion

Integration of health services is key to universal health coverage and an effective health system and makes the most of human and other resources. Experience with IMCI shows that the integration of monitoring and evaluation systems is often neglected. The global context is once more favourable for integration, including of monitoring and evaluation, particularly for child health. Countries now need to include monitoring and evaluation to produce a single health management information system, with a single set of indicators and training and which supports a team of data specialists. This would facilitate the reporting, analysis, and use of data for decision making. Global partners need to support these efforts and fully accept single national reporting systems. These principles should be incorporated into the redesign of IMCI programmes.

**Key messages**

- Integration of health services often neglects monitoring and evaluation systems, with negative consequences for both health workers and service delivery
- Global partners need to fully support single child health national reporting systems
- More investment is needed in staff to collect, report and analyse data
- Countries must develop standardised forms and indicators across health programmes
- New technologies can improve collection analysis and use of child health data

## Notes

Contributors and sources: This article is based on findings from a global review of Integrated Management of Childhood Illness (IMCI) commissioned by the WHO and funded by the Bill and Melinda Gates Foundation and three additional case studies. TD conceived the outline of this paper and drafted the first version, KR revised the paper and drafted the conclusions with inputs from EM, IM, HN, KMA, BN and KPO’N. BM created fig 1. All authors contributed intellectual content, edited the manuscript and approved the final version for submission.

Provenance and peer review: Commissioned; not externally peer reviewed.

This article is part of a series proposed by the South African Medical Research Council and based on findings from a global review of Integrated Management of Childhood Illness (IMCI) funded by the Bill and Melinda Gates Foundation. The BMJ peer reviewed, edited, and made the decision to publish the article with no involvement from the Medical Research Council. Open access fees were funded by the Health Systems Research Unit, South African Medical Research Council.

## References

[1] Frieden TR Six components necessary for effective public health program implementation. Am J Public Health 2014;104:17-22. 10.2105/AJPH.2013.301608 24228653 PMC3910052

[2] Frankel N, Gage A. M&E fundamentals: a self-guided mini-course. 2016. Measure evaluation. MS-07-20. https://www.globalhealthlearning.org/course/m-e-fundamentals

[3] Peersman G, Rugg D. Basic terminology and frameworks for monitoring and evaluation. 2004. http://www.unaids.org/sites/default/files/sub_landing/files/7_1-Basic-Terminology-and-Frameworks-MEF.pdf

[4] United Nations. The sustainable development goals report. 2016. https://unstats.un.org/sdgs/report/2016/

[5] Gove S The WHO Working Group on Guidelines for Integrated Management of the Sick Child Integrated management of childhood illness by outpatient health workers: technical basis and overview. Bull World Health Organ 1997;75(Suppl 1):7-24. 9529714 PMC2486995

[6] Dalgish SL Methods for the strategic review of IMCI and iCCM programmes. BMJ 2018;362:k2989 10.1136/bmj.k2989. 30061099 PMC6063343

[7] Boschi-Pinto C Labadie G Ramachandran DT Global implementation of the integrated management of childhood illness—twenty years on. BMJ Open 2018:8:e01907 10.1135/bmj-open.2017-01907 PMC6067364 30061428

[8] World Health Organization. IMCI indicators, monitoring and evaluation. Integrated Management of Childhood Illness (IMCI). 1999. http://apps.who.int/iris/bitstream/10665/65002/12/WHO_CHS_CAH_98.1K_eng.pdf

[9] Gage A, Ali D, Suzuki C. A guide for monitoring and evaluating child health programs. 2005. https://www.measureevaluation.org/resources/publications/ms-05-15

[10] Phalkey RK Yamamoto S Awate P Marx M Challenges with the implementation of an Integrated Disease Surveillance and Response (IDSR) system: systematic review of the lessons learned. Health Policy Plan 2015;30:131-43. 10.1093/heapol/czt097 24362642

[11] Lukwago L Nanyunja M Ndayimirije N The implementation of Integrated Disease Surveillance and Response in Uganda: a review of progress and challenges between 2001 and 2007. Health Policy Plan 2013;28:30-40. 10.1093/heapol/czs022 22669899 PMC3538461

[12] Unicef. Indicator and monitoring framework for the global strategy for women's, children's and adolescent's health (2016-2030). 2016. https://data.unicef.org/resources/indicator-monitoring-framework-global-strategy-womens-childrens-adolescents-health/

[13] Every Woman Every Child. Country data, universal accountability. monitoring priorities for the global strategy for women's, children’s and adolescent’s health (2016-2030). 2016. http://www.who.int/life-course/partners/global-strategy/gs-monitoring-readiness-report.pdf

[14] Maternal and Child Health Integrated Program (MCHIP). Indicator guide: monitoring and evaluating integrated community case management. 2013. http://1rqxbs47ujl4rdy6q3nzf554.wpengine.netdna-cdn.com/wp-content/uploads/2016/07/iCCM-Indicator-Guide.pdf

[15] World Health Organization. 2018 Global reference list of 100 core health indicators. 2018. http://www.who.int/healthinfo/indicators/2018/en/

[16] Health Data Collaborative. Better data. Better health. 2015. https://www.healthdatacollaborative.org/what-we-do/

[17] Atun R de Jongh T Secci F Ohiri K Adeyi O Integration of targeted health interventions into health systems: a conceptual framework for analysis. Health Policy Plan 2010;25:104-11. 10.1093/heapol/czp055 19917651

[18] Kasolo F Yoti Z Bakyaita N IDSR as a platform for implementing IHR in African countries. Biosecur Bioterror 2013;11:163-9. 10.1089/bsp.2013.0032 24041192 PMC3779000

[19] Reynolds HWSE Sutherland EG A systematic approach to the planning, implementation, monitoring and evaluation of integrated health services. BMC Health Serv Res 2013;13:168. 10.1186/1472-6963-13-168 23647799 PMC3649924

[20] Kizito K Adeline K Jean Baptiste K, editors. TRACnet: a national phone-based and web-based tool for the timely integrated disease surveillance and response in Rwanda. Online J Public Health Inform 2013;5:e202 10.5210/ojphi.v5i1.4598

[21] Guyon A Bock A Buback L Knittel B Mobile-based nutrition and child health monitoring to inform program development: an experience from Liberia. Glob Health Sci Pract 2016;4:661-70. 10.9745/GHSP-D-16-00189 28031303 PMC5199181

[22] Negandhi P Chauhan M Das AM Sharma J Neogi S Sethy G Computer tablet-based health technology for strengthening maternal and child tracking in Bihar. Indian J Public Health 2016;60:329-33. 10.4103/0019-557X.195868 27976658

[23] Kiberu VM Matofu JK Makumbi F Kyozira C Mukooyo E Wanyenze RK Strengthening district-based health reporting through the district health management information software system: the Ugandan experience. BMC Med Inform Decis Mak 2014;14:40. 10.1186/1472-6947-14-40. PMC4030005 24886567

[24] Kariuki JM Manders EJ Richards J Automating indicator data reporting from health facility EMR to a national aggregate data system in Kenya: an interoperability field-test using OpenMRS and DHIS2. Online J Public Health Inform 2016;8:e188. 10.5210/ojphi.v8i2.6722 28149444 PMC5266757

[25] Garley A Eckert E Sie A Strengthening individual capacity in monitoring and evaluation of malaria control programmes to streamline M&E systems and enhance information use in malaria endemic countries. Malar J 2016;15:300. 10.1186/s12936-016-1354-y 27233243 PMC4884432

[26] Bell JS Marais D Participatory training in monitoring and evaluation for maternal and newborn health programmes. Glob J Health Sci 2014;7:192-202. 10.5539/gjhs.v7n2p192 25716377 PMC4796430

